# Carduus Marianus

**Published:** 1891-05

**Authors:** H. Kunze


### CARDUUS MARIANUS.

DR. H. KUNZE.

(Zeitschrift des Berliner Hom. Aerzte, Vol. IX, 4, 5.)

In the works of the old school this beautiful plant is hardly mentioned, and even in our own school it is too much neglected, except by those who are acquainted with the works of Rademacher. The chief action of Carduus Mar. extends to diseases of the liver, bile, and spleen, and different consensual affections based on organic diseases of the same, as asthma, cough with pleuritic pains, local rheumatisms, especially of the intercostal muscles, and of the abdominal muscles, or peritoneum, also gastro-intestinal catarrh and dyspepsia. It has a decided action on the venous system, especially when based on hyperæmia of the liver or on hyperæmia by stasis in the portal system; in fact, this drug shows a specific relation to the vascular system. Epistaxis, menorrhagia, hæmorrhoids, hæmatemesis, and venous ulcers of the lower extremities were several times cured by it.

The first and most important indication for Carduus Mar. is hyperæmia of the liver, of the biliary ducts, of the portal circulation, uterus. In hyperæmia of the liver it suits acute and chronic cases, and we meet here, often, more or less swelling and painfulness of the right hypochondrium, with pressing, hammering, stitching pains on the right side under the short ribs, extending to the spine, or radiating through the chest to the right shoulder. Clinically may be mentioned that Carduus cured hepatic affections with great painfulness, though no swelling could be made out. There is a tendency to deep breathing, but this aggravates the pain; also worse by motion. In very acute cases this acute hepatic hyperæmia may be diagnosed as bilious fever, or acute hepatitis, or typhlitis, or be mistaken for a puerperal peritonitis or as a spurious pneumonia.

Chronic hepatic hyperæmia is often accompanied by chronic pleuritic stitches in the left and right hypochondrium, belly-ache in the cœcal region, with emaciation, dirty-yellow color of the face, hectic fever; sometimes hæmorrhages set in, as epistaxis; bloody sputa and hæmatemesis, metrorrhagia, or ischias and intercostal muscular pains. Gastro-intestinal catarrh or jaundice may complicate the case, and the indications for Carduus are: dull headache, especially in forehead and temples, obtuseness of head and vertigo, nose bleed, bitter, pappy, flat taste, eructations, waterbrash, white tongue, especially in the centre, with red tip and edges, or only on one side; sometimes vomiting of an acid, green fluid. Stools are at first mostly brown and consistent; neither constipation nor diarrhœa, later light-yellow, mushy or diarrhœic. The urine is at first bright-yellow, becomes brownish from the addition of biliary pigment, mostly alkaline or sour, depositing a cloudy sediment. The gastro-intestinal catarrh is subacute or a status-gastricus or a gastralgia with constricting pains and at their acme vomiting, cold risings from præcordium to the throat, and ending with the sensation of constriction in the throat. Carduus helped sometimes in the vomiting of pregnancy, when it takes place mornings, on an empty stomach, is watery and not tasting after food. Some praise it in biliary colic from gall-stones, but *post hoc* is not always *propter hoc*, though it stops the vomiting.

Melancholia, in consequence of hepatic troubles, may yield to Carduus; when accompanied by cough, which is either dry or mucous, with blood-streaks or blood; mornings, difficult expectoration of thick, yellow sputa; evening, fever and stitching pains in the side. Some patients complain of dyspnœa, so that without an examination one might think of pleuritis or pneumonia.

In gastralgia, Carduus Mar. is entirely too much neglected. When the pains are constricting with vomiting at the acme of the attack, and cold spasmodic constriction rising from the stomach to the œsophagus, or a pressing, stitching pain in the right hypochondrium, radiating into the back or shoulder. Such gastralgiæ are often merely nervous; yielding to Nux-vomica, or according to indications to Carduus.

In chronic hyperæmia of the spleen, its indications are: chronic stitches in the left hypochondrium, hæmatemesis, intermittent fever and intermittent neuralgia. Such a state may be a sequela of typhoid fever or malaria, and yields promptly to this remedy. It was formerly considered a valuable remedy in intermittents. Pulmonary hæmorrhages in connection with hepatic troubles cannot be cured by so-called hepatic remedies, but yield to Carduus. The same results happen in coughing up blood from spleen troubles; where the patient is relieved when lying on left side. Acute and chronic bronchial catarrh, acute and chronic angina in connection with affections of the liver or spleen, even asthma may yield to this good remedy. Carduus Marianus ought to be thought of in hæmorrhages, even when there are no hepatic or splenetic troubles. Prof. Rapp recommends it highly for habitual epistaxis; which in young persons appears as a symptom of psora, and differs herein from Bryonia, Hamamelis, or Crocus. It is especially effective in uterine hæmorrhages, which are too frequently not idiopathic uterine affections, but are caused by affections of the liver, spleen, or kidneys.

Windelband cured one hundred and fifteen cases (out of one hundred and ninety-six) of varicose ulcers of the legs with Carduus. The ulcers showed a bluish, browned color, surrounded by dilated varicose veins, with callous, indented edges; easily bleeding after an injury, bursting of a varix, often preceded by an eczema more rarely after an inflammation of the connective tissue, and mostly emanating from the scratching of the itching eczematous skin. The pains were mostly moderate, sometimes the patients complained of burning in the ulcers, and around them, especially during the healing process.

Carduus is specific in localized muscular rheumatism, whether in the abdominal muscles, in the hip, thigh down to the ankles, or under the short ribs or sacrum, especially where hepatic symptoms prevail. The abdominal pains may be so severe that one thinks of a peritonitis. Cases from practice illustrate these applications of Carduus Marianus, and they certainly deserve a more thorough study of this too often neglected remedy.

